# Deciphering the Tenascin-C Nexus: A Comprehensive Review of Its Involvement in Chronic Respiratory Diseases

**DOI:** 10.3390/pathophysiology32030044

**Published:** 2025-09-08

**Authors:** Juan Manuel Velázquez-Enríquez, Jovito Cesar Santos-Álvarez, Karina González-García, Itayetzi Reyes-Avendaño, Víctor Acevedo-Sánchez, Ariadna Jalife Gómez, Antonio Arcos-Román, Jaime Arellanes-Robledo, Verónica Rocío Vásquez-Garzón, Rafael Baltiérrez-Hoyos

**Affiliations:** 1 Centro de Investigación en Nutrición y Alimentación, Licenciatura en Nutrición, Universidad del Istmo, Carretera Transísmica Juchitán, La Ventosa km. 14, La Ventosa 70102, Oaxaca, Mexico; 2 Laboratorio de Fibrosis y Cáncer, Facultad de Medicina y Cirugía, Universidad Autónoma Benito Juárez de Oaxaca, Ex Hacienda de Aguilera S/N, Sur, San Felipe del Agua 68020, Oaxaca, Mexico; jovitocesarsa@cecad-uabjo.mx (J.C.S.-Á.); k.igg@cecad-uabjo.mx (K.G.-G.); itayetzi.reyes94@cecad-uabjo.mx (I.R.-A.); vrvasquezga@secihti.mx (V.R.V.-G.); 3 Instituto de Salud Pública, Universidad Veracruzana (UV), Xalapa 91190, Veracruz, Mexico; vicacevedo@uv.mx; 4 Escuela de Ingeniería y Ciencias, Tecnológico de Monterrey, Campus Querétaro, Av. Epigmenio González, No. 500 Fracc. San Pablo, Santiago Querétaro 76130, Querétaro, Mexico; a01369674@tec.mx; 5 Laboratorio de Diagnóstico Molecular, Facultad de Medicina, Universidad Autónoma de Guerrero, Avenida Solidaridad S/N Hornos Insurgentes, Acapulco 39300, Guerrero, Mexico; arcosan@gmail.com; 6 Laboratorio de Enfermedades Hepáticas, Instituto Nacional de Medicina Genómica, Periférico Sur No. 4809, Col. Arenal Tepepan, Alcaldía Tlalpan, D.F., México City 14610, Mexico; jarellanes@inmegen.gob.mx; 7 SECIHTI-Facultad de Medicina y Cirugía, Universidad Autónoma Benito Juárez de Oaxaca, Ex Hacienda de Aguilera S/N, Sur, San Felipe del Agua 68020, Oaxaca, Mexico

**Keywords:** chronic pulmonary diseases, pulmonary diseases, lung disease, tenascin-C, extracellular matrix protein, inflammation, fibrosis

## Abstract

Tenascin-C (TNC) is an extracellular matrix (ECM) protein with key roles in various biological processes, such as embryonic development and tissue regeneration. However, its deregulated expression can contribute to pathological responses, promoting chronic inflammation, fibrosis, or tumor progression. It belongs to the tenascin family, a class of extracellular proteins that interfere with cellular events in both physiological and pathological contexts, interacting specifically with cells and other components of the ECM. TNC has emerged as a key player in the pathogenesis of chronic respiratory diseases (CRDs), including asthma, chronic obstructive pulmonary disease (COPD), lung cancer (LC), pulmonary hypertension (PH), and idiopathic pulmonary fibrosis (IPF). The influence of TNC on cellular responses, which is mediated by precise interactions with cellular receptors and ligands, triggers complex intracellular signaling cascades associated with the inflammatory response, fibrosis, and tumorigenesis in these CRDs. This review synthesizes recent evidence highlighting the multifaceted roles and underlying mechanisms of TNC in the context of these CRDs.

## 1. Introduction

Chronic respiratory diseases (CRDs) or chronic pulmonary diseases are a group of pathologies that affect different lung structures [1,2]. This group of diseases remains a significant public health problem worldwide, representing the third leading cause of death globally, surpassed only by cardiovascular diseases and tumors. They are, therefore, a significant cause of morbidity and mortality worldwide [1,2,3]. A systematic analysis of data from the 2017 Global Burden of Disease (GBD) study estimated the prevalence and mortality attributable to CRDs from 1990 to 2017. Approximately 544.9 million people worldwide suffered from these diseases in 2017, representing an increase of 39.8% compared with the number affected in 1990. Compared with those in 1990, the global prevalence and mortality of CRDs in 2017 increased by approximately 7.1% and 18%, respectively [1]. Therefore, due to the significant impact that CRDs such as asthma, chronic obstructive pulmonary disease (COPD), lung cancer (LC), pulmonary hypertension (PH), and idiopathic pulmonary fibrosis (IPF) have on global health, it is essential to study the biological context involved in their development, since these conditions not only differ in their epidemiology and in certain specific molecular and cellular mechanisms that constitute true distinctive “hallmarks” of each one, but also share common pathophysiological pathways that jointly contribute to their progression [1,2,4,5,6,7,8]. Understanding these processes is essential for the identification of early biomarkers, the discovery of novel therapeutic targets, and the development of more effective and personalized treatment strategies that can improve patient outcomes and reduce the global burden of CRDs [2,7,8].

Tenascin-C (TNC) belongs to the tenascin family, a group of extracellular matrix (ECM) glycoproteins composed of four prominent members: TNC, tenascin-R (TNR), tenascin-X (TNX), and tenascin-W (TNW) [9,10]. Although all tenascins share standard structural features, such as fibronectin type III (FNIII) domains and trimer/hexamer structures, they differ significantly in their tissue expression pattern and biological functions [10]. Among them, TNC is considered the founding member of this protein family and has been the most widely studied due to its dynamic re-expression during pathological processes, including inflammation, fibrosis, and tumorigenesis. At the same time, the other tenascins have more restricted functions [9,10,11]. For example, TNR is expressed mainly in the central nervous system, TNX is involved in connective tissue integrity, and TNW has recently been associated with specific tumor processes, including the promotion of tumor cell proliferation, the enhancement of angiogenesis, and the facilitation of metastatic spread in cancers such as glioblastoma and breast cancer [10]. This functional versatility and its inducible expression have positioned TNC as a key molecule in the research on various diseases, including cardiac disease [12], rheumatic diseases [13], and multiple types of cancers [14]. Recent evidence has aroused particular interest because of its possible involvement in respiratory conditions, such as asthma [15], LC [16], PH [17], COPD [18], and IPF [19], where it has been shown to actively participate in tissue remodeling, cellular activation, and the amplification of the inflammatory response. This review presents the current evidence available on the role of TNC in these CRDs.

## 2. TNC: Structure

**Figure 1:**
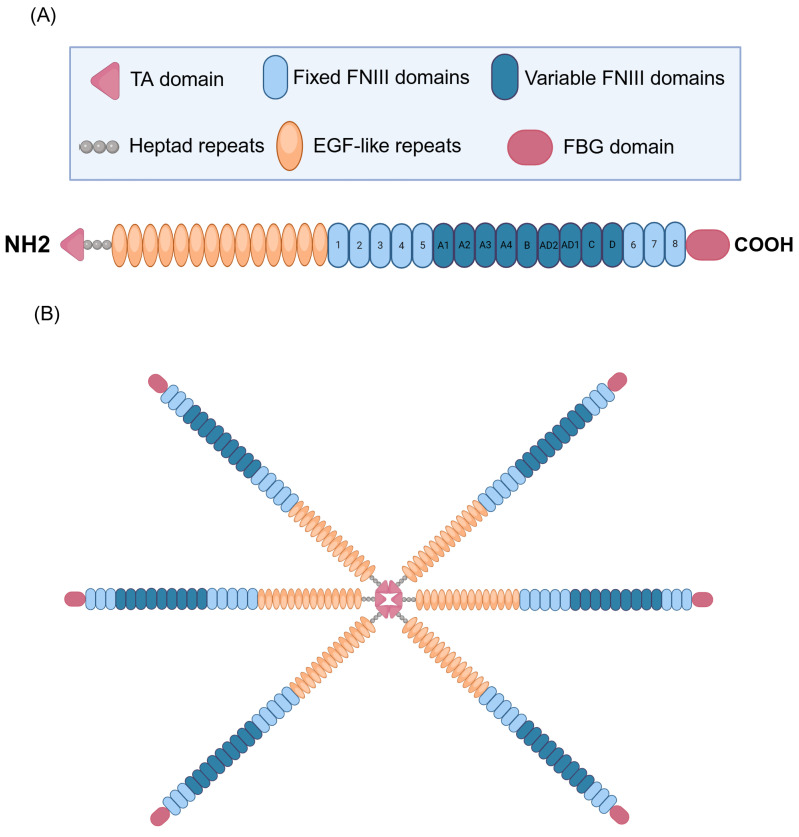
Structure of human tenascin C (TNC). (**A**) Representation of the TNC subunit. The N-terminal end contains the tenascin assembly domain (TA). The structure includes 14 EGF (epidermal growth factor-like) repeats, essential for cell signaling and the modulation of the surrounding microenvironment. In addition, it contains 17 fibronectin III (FNIII)-like repeats, divided into 8 fixed and 9 variable FNIII domains, which gives it flexibility and structural adaptability, allowing for specific interactions with various cellular and ECM components. A distinctive feature is the presence of a fibrinogen-like globular (FBG) domain at the C-terminus, which amplifies the ability of TNC to participate in pathological processes such as inflammation and ECM tissue remodeling. In addition, TNC possesses heptad repeats, which are crucial for forming the functional hexamer of the protein, contributing to its structure and biological activity. (**B**) Representation of TNC in its hexameric form. Created with BioRender.com.

TNC, a multifaceted glycoprotein belonging to the tenascin family of proteins, displays an intricate and modular structure that supports its diverse biological functions in the context of CRDs [16,19,20]. The structural complexity of TNC originates from its modular composition, which frequently adopts the form of hexamers with a molecular weight of approximately 220–400 kDa [21,22]. Each monomer has four distinct domains, each performing specific functions. It contains a tenascin assembly (TA) domain at the N-terminal end and has heptad repeats, which are essential for the formation of the functional hexamer of the protein (Figure 1). In addition, it contains 14 epidermal growth factor-like (EGF-like) repeats, which play crucial roles in cell signaling and the modulation of the surrounding microenvironment [21,23,24,25]. It also contains 17 fibronectin type III (FNIII)-like repeats, which are divided into eight fixed FNIII domains and nine variable FNIII domains. These FNIII domains contribute to the flexibility and structural adaptability of TNC, allowing it to interact specifically with various cellular and ECM components [23,26,27,28]. A distinctive feature is the presence of a fibrinogen-like globular (FBG) domain at the C-terminus, which amplifies the ability of TNC to participate in various pathological processes and modulate different pathophysiological events, including inflammation and tissue remodeling of the ECM [23,29,30]. This modular architecture allows TNC to be versatilely involved in various physiological and pathological processes in the respiratory context.

## 3. TNC: Expression, Localization, and Regulation

The specific distribution of TNC in the respiratory system does not follow a uniform pattern but is tightly regulated by the tissue’s microenvironmental characteristics and physiological state [31]. In healthy lungs, TNC predominantly localizes to regions undergoing dynamic remodeling, such as sites of branching morphogenesis during development or areas of repair and regeneration in response to injury. This precise targeting underscores the fundamental role of TNC as an essential molecular modulator in tissue architecture, actively participating in processes critical to maintaining lung homeostasis and repair [31,32].

**Figure 2:**
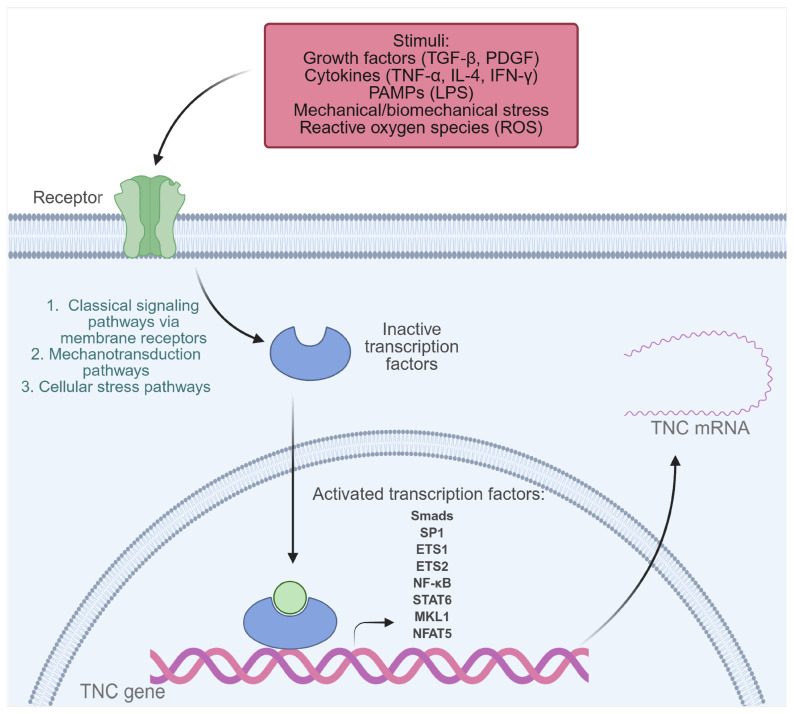
Transcription factors activated by extracellular stimuli that induce TNC expression in pathological conditions. Transcription factors that play a key role in TNC gene expression include NF-κB, Smads, SP1, ETS1, ETS2, STATs, MKL1, and NFAT5. These are activated in response to various inflammatory or pathophysiological stimuli, such as growth factors (TGF-β, PDGF), cytokines (TNF-α, IL-4, IFN-γ), PAMPs (such as LPS), mechanical or biomechanical stress, and reactive oxygen species (ROS). Created with BioRender.com.

Under normal physiological conditions, TNC is expressed to a limited extent in adult tissues. However, its synthesis can be transiently activated in the face of inflammation or tissue injury, a process that depends on the activation of multiple transcription factors and signaling pathways capable of integrating mechanical, immunological, infectious, and developmental stimuli (Figure 2) [21,23,31,33]. Among the identified regulators are Nuclear Factor Kappa B (NF-κB), activated by LPS, and tumor necrosis factor-alpha (TNF-α) in human monocytes and chondrocytes, as well as STATs, activated by IL-4, and Interferon gamma (IFN-γ) through the Janus kinase/signal transducer and activator of transcription (JAK-STAT) pathway [33,34,35,36]. Under mechanical stress conditions, activation of NFAT5 and MKL1 has been observed, which promote TNC expression in vascular smooth muscle cells (VSMCs) and the mouse fibroblast cell line NIH3T3, respectively. Likewise, biomechanical stress stimulates the NF-κB pathway through the generation of reactive oxygen species (ROS) in neonatal cardiac myocytes, resulting in increased TNC expression [37,38,39]. Furthermore, growth factors such as transforming growth factor beta (TGF-β) induce TNC through Smad3/4 signaling, and platelet-derived growth factor (PDGF) acts through the phosphoinositide 3-kinase (PI3K)/Akt pathway, promoting the binding of Sp1, Ets1, and Ets2 to the TNC promoter in human dermal fibroblasts [40,41].

Homeobox factors such as Prx1, Pou3F2, Otx2, and Evx-1 also modulate TNC expression in developmental and tissue-specific patterns [23,33]. Prx1, for example, enhances TNC transcription in vascular smooth muscle cells, while Otx2 exerts a repressive effect [23,42]. The Notch2/RBPJk pathway has been identified as a direct regulator of TNC in specific tumor contexts [43]. Finally, conserved elements in the TNC promoter, such as the TATA box, and multiple binding sites for factors such as Sp1, AP-1, AP-2, C/EBP, Krox-20, and NF-1 allow for a precise spatiotemporal regulation of its expression [33,44].

These data show how TNC transcription can be activated or repressed by different stimuli, including pathogen-associated molecular patterns (PAMPs), cytokines, mechanical stress, ROS, and growth factors. This precise control allows TNC to participate in both physiological processes—such as embryonic development and tissue repair—and pathological states, including fibrosis, cancer, and chronic inflammatory diseases.

## 4. Cellular Receptors and Ligands of TNC

**Figure 3:**
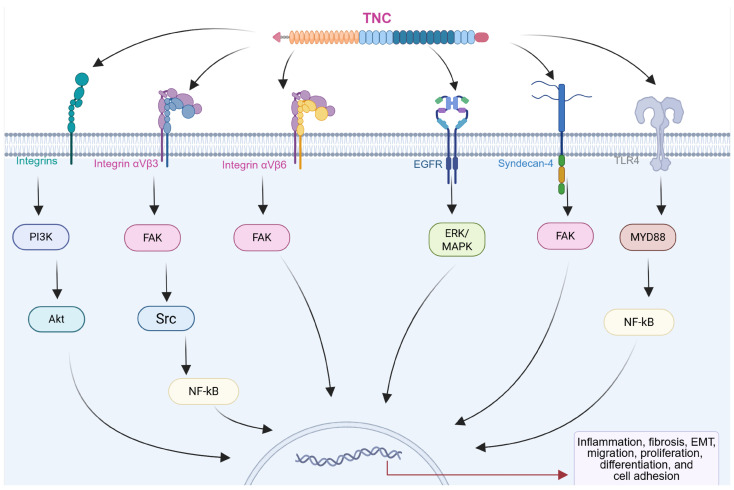
Diagram showing the interactions of TNC with different receptors on the cell surface. Activation of integrins, epidermal growth factor receptor (EGFR), syndecan-4, and toll-like receptor 4 (TLR4) triggers the activation of intracellular effectors such as focal adhesion kinase (FAK) and YAP, which induce changes in gene transcription, resulting in modifications in the expression of proteins associated with cellular processes in multiple chronic diseases. Created with BioRender.com.

The biological functions of TNC are intricately linked to its interactions with cellular receptors and ligands, generating a complex network that modulates cellular behaviors and influences the pathophysiology of chronic diseases, including CRDs (Figure 3) [14,45,46]. Integrins, a family of transmembrane receptors, play critical roles as primary mediators of TNC-induced signaling. TNC features conserved binding sites for specific integrin subtypes, such as α2β1, αvβ6, αvβ1, αvβ3, α7β1, α8β1, and α9β1 [14,47,48]. These interactions occur primarily through the FNIII repeat domains of TNC, which contain specific integrin-recognition motifs such as AEIDGIEL and LDV, depending on the isoform [14]. The interaction of these integrins with TNC triggers intracellular signaling pathways, including those involving focal adhesion kinase (FAK) and PI3K/Akt, which result in changes in cell adhesion, migration, and the activation of subsequent pathways. This process is essential for regulating cellular responses in the pathogenesis of chronic diseases such as fibrosis and cancer in different organs [14,47,48,49].

For example, in murine models of angiotensin II-induced hypertensive cardiac fibrosis, TNC promotes collagen deposition, macrophage recruitment, and the upregulation of inflammatory cytokines. These profibrotic and inflammatory responses, mediated by the αvβ3/FAK-Src/NF-κB pathway, were markedly reduced in TNC knockout mice, highlighting the key role of TNC in modulating cardiac fibrosis [48]. TNC has also emerged as a crucial biomarker and mediator in renal fibrosis. At the cellular level, TNC affects tubular cell integrity by inducing partial EMT, and mechanistically, TNC activates αvβ6 integrins and FAK. Blockade of these pathways reversed the EMT and alleviated renal fibrosis, suggesting that the TNC/integrin αvβ6/FAK signaling cascade plays an important role in renal fibrosis [50]. Moreover, a study revealed that TNC drives cancer cell migration through an intrinsic signaling mechanism, acting within the tumor cells themselves. Using a murine xenograft model of advanced human osteosarcoma, TNC and its integrin α9β1 receptor were determined to be crucial for lung metastasis. Activation of this pathway reduces the autonomous expression of Yes-associated protein (YAP) transcription factor target genes in tumor cells. These findings highlight the role of TNC in invasive migration and metastatic progression [47].

In addition to integrins, TNC interacts with various cell membrane receptors, expanding its repertoire of cellular interactions. These include syndecan, epidermal growth factor receptor (EGFR), and toll-like receptors (TLRs), which contribute to the modulation of inflammatory responses and the activation of signaling pathways associated with tissue remodeling and repair [14,29,30,51,52,53]. TNC also interacts with EGFR through its EGF-like repeats, promoting cell proliferation. This interaction activates the extracellular signal-regulated kinase/mitogen-activated protein kinase (ERK/MAPK) signaling pathway, which is involved in cell growth and differentiation [53].

TNC has been shown to activate toll-like receptor 4 (TLR4), triggering NF-κB-dependent signaling that promotes proinflammatory and profibrotic responses. In fibroblasts and myofibroblasts, this pathway induces cytokine production (e.g., IL-6, TNF-α, and IL-8) and collagen synthesis, while its inhibition blocks these effects. These findings establish TNC as a key modulator of inflammation and fibrosis via TLR4 signaling [29,30,51].

In addition, the ability of TNC to interact with various ECM proteins, including FN, has been documented [22]. In addition to its affinity for ECM components, TNC has the remarkable ability to bind multiple growth factors, including members of the PDGF family, fibroblast growth factor (FGF) family, TGF-β superfamily, and insulin-like growth factor-binding proteins (IGF-BP) [26,54]. Its ability to bind multiple growth factors suggests that these interactions are not merely structural but could modulate the availability and local activity of growth factors, affecting cellular signaling and the progression of pathological processes [26]. For example, in breast cancer models, TNC favors the survival and proliferation of pulmonary micrometastases by influencing stem cell-associated signaling pathways. Although the specific binding of growth factors to TNC still requires further study, the identification of interaction domains opens new opportunities to explore its functional role in different diseases [26,55,56].

The binding of TNC to its receptors activates multiple signaling pathways, including MAPK, PI3K/Akt, and FAK pathways. These signaling cascades contribute to cellular responses such as cell proliferation, migration, adhesion, and survival [49,50,52,53]. The integration of TNC-mediated signaling with other cellular processes further highlights its role as a central regulator of cellular behavior in the respiratory microenvironment.

Furthermore, TNC exhibits remarkable binding capacity to heparin and the heparan sulfate (HS) chains of syndecans, broadening its spectrum of molecular interactions [26,57,58]. In different cellular contexts, such as fibroblasts and tumor cells, TNC exerts a marked anti-adhesive effect by interfering with fibronectin (FN)-mediated adhesion signaling. This effect is attributed to TNC ability to block the interaction of the HepII/syndecan-4 site of FN by directly binding to the FNIII13 domain. As a consequence, a reduction in cell adhesion and proliferation is observed, associated with altered FAK phosphorylation. However, overexpression of syndecan-4 or reintroduction of the FNIII13 peptide can restore adhesion and proliferation, revealing a key mechanism by which TNC modulates the adhesive function of FN and interferes with syndecan-4/integrin co-signaling [52,59].

In general, TNC can bind to multiple ECM proteins and various cellular receptors, which gives it great functional versatility and the potential to modulate different pathways and cellular processes associated with pathologies [9,14,23]. However, its biology and functions are not yet fully elucidated, and its effects appear to be closely dependent on the cellular and microenvironmental context. For example, in cancer, TNC exhibits a dual role in the regulation of cell adhesion and proliferation. On the one hand, it can interact with integrins, activating signaling pathways such as FAK/Src and PI3K/AKT, which promote tumor cell adhesion, proliferation, and survival, favoring tumor progression and metastasis [14,47,49]. On the other hand, TNC can also exert anti-adhesive effects by interfering with fibronectin-mediated signaling, particularly through its binding to the FNIII13 domain and the inhibition of the coreceptor function of syndecan-4. This mechanism reduces focal adhesion signaling and cell proliferation [59]. This apparent paradox highlights the modulatory nature of TNC, which acts not exclusively as a stimulatory or an inhibitory factor but also as a complex regulator in pathologies such as cancer.

## 5. Common Pathogenic Mechanisms in Chronic Respiratory Diseases: A Framework for Understanding the Role of TNC

Despite their etiological differences, CRDs such as IPF, COPD, PH, LC, and asthma converge on a set of standard cellular and molecular mechanisms. These mechanisms involve a sequence of interconnected events that begin with epithelial and/or endothelial injury and culminate in tissue remodeling, fibrosis, or malignant transformation [7,8,60,61,62,63,64,65,66]. Understanding this shared pathogenic core provides a unified framework to explore the multifaceted role of TNC across distinct disease contexts.

At the initial stage of disease development, epithelial or endothelial cell activation occurs in response to external or internal insults. These triggers include PAMPs, cytokines, growth factors, and mechanical stress [67,68,69,70,71]. The activated epithelial and endothelial layers subsequently initiate a cascade of signaling events, prominently involving the NF-κB and JAK/STAT pathways, as well as additional pathways such as TGF-β/Smad, phosphoinositide 3-kinase/protein kinase B (PI3K/Akt), mitogen-activated protein kinases (MAPKs). Additionally, oxidative stress and the generation of ROS act as amplifiers of these pathways, enhancing cellular damage and inflammation [67,68,69,70].

Among the central downstream events in CRDs are epithelial-to-mesenchymal transition (EMT) and its endothelial counterpart, endothelial-to-mesenchymal transition (EndoMT). EMT is a biological process in which epithelial cells lose their polarity and adhesion properties and acquire mesenchymal features such as motility, contractility, and ECM production ability. Similarly, EndoMT allows endothelial cells to transition into mesenchymal phenotypes, contributing to vascular remodeling and fibrosis. Both processes are key contributors to the generation of activated fibroblasts and myofibroblasts—effector cells that orchestrate tissue remodeling, fibrosis, and in some contexts, tumor progression [66,72,73,74].

EMT is classically categorized into three subtypes: type I (associated with development), type II (related to tissue regeneration and fibrosis), and type III (linked to tumor progression and metastasis) [75]. In the context of CRDs, type II EMT has been observed in alveolar epithelial cells in IPF [76], while EndoMT contributes to vascular remodeling in PH [77]. In airway diseases such as COPD and asthma, bronchial epithelial cells—particularly basal cells—undergo EMT in response to chronic inflammation and environmental insults [66,78]. In LC, EMT facilitates epithelial plasticity, invasion, and resistance to therapy [79].

These transitions are primarily regulated by a network of signaling pathways, including TGF-β/Smad, BMP, Wnt/β-catenin, PI3K/Akt, and MAPKs, in addition to inflammatory mediators (e.g., IL-1β, TNF-α), ROS, hypoxia, and mechanical stress [66,73,74,80]. Notably, TNC has been implicated in the regulation of EMT and EndoMT by modulating cell–matrix interactions, increasing the sensitivity to profibrotic signals, and altering the transcriptional profile of transitioning cells [45,81,82,83,84,85,86]. These effects not only promote the progression of fibrotic processes but also may contribute to malignant cell transformation by facilitating the invasion, migration, and phenotypic plasticity characteristic of tumor development [14,45,81,82,83,84,85].

Thus, within this pathogenetic framework, TNC emerges as a multifunctional element that acts at multiple levels. Its expression has been associated both with a secondary marker of tissue damage and with an active mediator of various pathological responses. TNC modulates interactions between cells and the ECM, promotes fibroblast migration, favors EMT, and modifies the immune response in a manner dependent on the biological context [14,23,45,51,81,87].

Therefore, we hypothesize that, during the development of each CRD, early events such as inflammation, ROS generation, and mechanical stress activate both the epithelium and the endothelium. These initial stimuli trigger transitional programs, such as EMT and EndoMT, followed by intracellular signaling cascades that promote the induction of TNC and other ECM components [19,67,68,69,72,74]. This progressive sequence culminates in pathological outcomes such as fibrosis, vascular remodeling, or tumor progression, depending on the specific cellular and tissue context [19,88,89]. In the following sections, we explore how TNCs are integrated into this pathogenic architecture in each CRD.

## 6. The Role of the TNC in Chronic Respiratory Diseases

**Figure 4:**
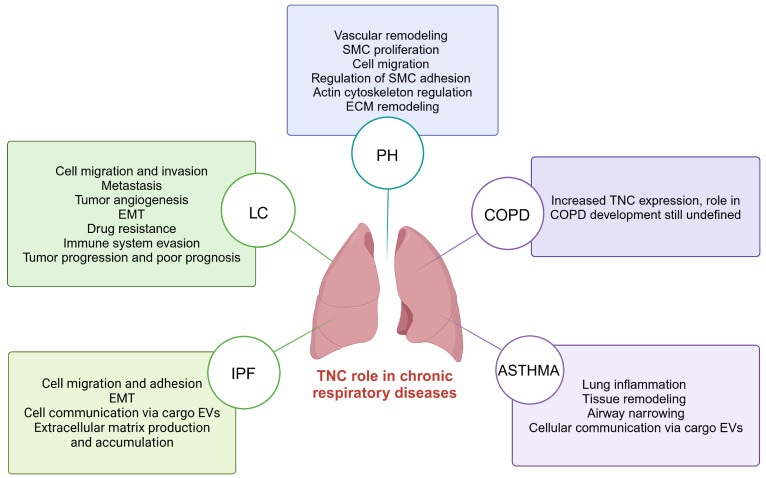
TNC-mediated processes in chronic respiratory diseases including IPF, LC, PH, COPD, and asthma. TNC; tenascin-C; IPF; idiopathic pulmonary fibrosis: LC; lung cancer; PH; pulmonary hypertension; COPD; chronic obstructive pulmonary disease. Created with BioRender.com.

Recent research suggests that TNC is key in developing CRDs, such as IPF, LC, PH, COPD, and asthma [19,90,91,92,93]. Increased TNC expression is observed in these conditions, suggesting its involvement in several pathological processes that lead to the development and progression of these lung diseases [45,46,94,95] (Figure 4).

### 6.1. TNC in Idiopathic Pulmonary Fibrosis

IPF is a chronic disease of somewhat uncertain etiology that causes progressive and irreversible lung damage, distinguished by increased scarring or fibrosis that replaces the healthy parenchymal tissue in the lungs, resulting in respiratory distress and failure that ultimately result in patient death [96,97]. The incidence of IPF increases with age, with IPF manifesting predominantly in individuals aged 65 years and older. The incidence and prevalence rates of IPF are estimated to range from 0.09 to 1.30 and from 0.33 to 4.51 per 10,000 persons, respectively, with a higher prevalence in men than in women [4].

Its development is based on the complex interaction between predisposing genetic factors, environmental exposures, and aberrant cellular processes [98]. Predisposing factors include genetic mutations in genes related to epithelial integrity and repair, such as TERT, TERC, and MUC5B. Furthermore, chronic environmental exposures—such as tobacco smoke, organic and inorganic dust, and a history of viral infections—act as triggers for alveolar damage [64,98]. The critical initial event is recurrent alveolar epithelial damage, primarily in alveolar type II (ATII) cells. These cells, after injury, present hyperplasia and altered function, releasing a range of profibrotic mediators and proinflammatory cytokines, including TGF-β1, PDGF, FGF, vascular endothelial growth factor (VEGF), TNF-α, and interleukins such as IL-1β and IL-6 [64,98,99]. Importantly, several of these mediators—particularly TGF-β1—induce EMT, a process in which injured epithelial cells acquire mesenchymal features. The activation of these mediators also induces the proliferation and activation of fibroblasts, as well as their differentiation into myofibroblasts, cells responsible for the synthesis and excessive accumulation of ECM. This abnormal deposition causes thickening of the alveolar walls, destruction of the capillary bed, and loss of lung elasticity. Simultaneously, alveolar macrophages and resident immune cells participate in the perpetuation of the inflammatory and fibrogenic process, secreting mediators that amplify epithelial damage and mesenchymal activation [98,99]. Fibrosis is maintained and progresses through the sustained activation of intracellular signaling pathways, primarily the SMAD pathway through TGF-β1 but also the Wnt/β-catenin, MAPK, PI3K/AKT, Rho/Rock, mTOR, and JAK/STAT pathways, which regulate altered epithelial apoptosis, aberrant cell proliferation, and the resistance to apoptosis of fibroblasts and myofibroblasts. This dysfunctional cycle of persistent epithelial damage, exacerbated mesenchymal activation, dysregulated immune response, and tissue remodeling leads to the irreversible formation of fibrous tissue, with progressive deterioration of lung function and an unfavorable prognosis [64,98,99,100].

In recent years, interest has increased in the role of ECM proteins associated with the pathological process of IPF. In this context, the association of TNC with IPF has been described in several studies, and interestingly, TNC was shown to be upregulated at the protein and mRNA levels in lung tissue from patients with IPF compared with lung tissue from healthy patients [19,101]. In addition, TNC expression at the mRNA and protein levels increases during the development of an experimental murine model of bleomycin-induced IPF, and this significant increase correlates with collagen formation [102]. TNC is involved in several biological processes associated with pulmonary fibrosis, such as fibroblast migration and cell adhesion, EMT, and deposition of ECM proteins, mainly collagen [19,51]. In addition, studies in a bleomycin-induced IPF murine model demonstrated that, compared with WT control mice, Tnc^−/−^ mice are protected against lung damage, exhibit decreased collagen accumulation, and show significantly reduced IPF development [45,51].

Compared with unstimulated fibroblasts, normal human lung fibroblasts cultured in the presence of TGF-β1, a key regulator of fibrotic processes that induces the differentiation of lung fibroblasts toward the myofibroblastic phenotype, presented significantly increased TNC mRNA expression and protein synthesis, and this increase was dose- and time-dependent following TGF-β1 exposure [19,102]. A recent proteomic analysis demonstrated that TNC is enriched in extracellular vesicles (EVs) secreted by lung fibroblasts with a human IPF phenotype compared with EVs released by normal human lung fibroblasts, suggesting that the essential role of TNC in the fibrogenesis processes associated with IPF might be closely associated with its release as part of the cargo of EVs [96]. In this context, available evidence suggests that TNC is involved in the development of IPF through processes such as cell adhesion and migration, EMT, EV-mediated cell communication, and ECM production and accumulation. TNC’s ability to enhance myofibroblast activation and promote ECM deposition makes it a key mediator in the perpetuation of the fibrotic cycle. However, significant gaps in knowledge regarding the specific role of TNC in IPF remain, which warrants further investigation in future studies.

### 6.2. TNC in Lung Cancer

LC, a malignant neoplasm originating in lung tissues, is classified into two main categories: small cell lung cancer (SCLC) and non-small cell lung cancer (NSCLC). NSCLC encompasses a variety of histologic subtypes and accounts for approximately 85% of LC cases [103,104]. LC poses a major global public health challenge. Its prevalence has reached alarming proportions, making it one of the leading causes of mortality today. It accounts for approximately 11.4% of all cancers and is the leading cause of cancer death worldwide, accounting for approximately 18.0% of all cancer deaths [1,5]. The disease is most strongly linked to tobacco use, which accounts for the majority of cases. Nevertheless, other factors—including environmental and occupational exposure to carcinogens (e.g., arsenic, asbestos, air pollutants), genetic susceptibility, and pre-existing CRD—also play important roles in its pathogenesis [105,106,107]. These risk factors contribute to a multistep process of carcinogenesis characterized by cumulative genetic and epigenetic alterations, disruption of normal cell cycle control, evasion of apoptosis, and progressive malignant transformation of lung epithelial cells [105,108,109].

At the molecular level, lung carcinogenesis results from the convergence of genetic alterations and environmental exposures, leading to the dysregulation of key cellular pathways that promote uncontrolled cell proliferation [109,110]. This process involves the activation of oncogenes (e.g., MYC, KRAS, EGFR, and ALK), the inactivation of tumor suppressor genes (e.g., TP53, RB1, and p16), and aberrant signaling through pathways such as EGFR, PI3K/AKT/mTOR, and JAK/STAT [79,109,110,111,112,113]. Chronic inflammation within the lung microenvironment further amplifies tumor progression through the release of cytokines (e.g., IL-1β, IL-4, IL-6, IL-11, IL-12, TGF-β, TNF-α), growth factors, and proteolytic enzymes, which drive angiogenesis, EMT, and ECM remodeling [79,114]. The tumor microenvironment (TME) is a highly dynamic and complex network composed of stromal cells, tumor-associated fibroblasts (TAFs), and diverse immune cell populations, including T cells, myeloid-derived suppressor cells, natural killer cells, mast cells, tumor-associated neutrophils (TANs), and tumor-associated macrophages (TAMs). These cellular components interact in a tightly regulated manner to sustain tumor growth, promote immune evasion, and enhance the metastatic potential. Additionally, persistent inflammation, oxidative stress, altered apoptosis, dysregulated angiogenesis, and aberrant cell activation converge to create a permissive niche that supports the aggressive clinical behavior of LC and contributes to its resistance to conventional therapies [79,114,115].

In this context, attention has focused on ECM proteins, whose role is essential for carcinogenic developmental progression. A prominent topic of current research is TNC, an ECM protein that plays a key role in the development of multicellular organisms, as well as in pathological processes such as inflammation, tissue injury, tumor angiogenesis, and metastasis [90,116]. In recent years, its significant role in the development of LC has been revealed [88,90]. TNC has been shown to be overexpressed at the mRNA and protein levels in tumor tissue from patients with NSCLC compared with adjacent normal tissue, and its expression is correlated with unfavorable clinical outcomes, larger tumor size, lymph node metastasis, and disease recurrence [46,88,90]. On the other hand, it has been postulated that the quantification of serum TNC levels could constitute a potentially predictive indicator of intratumoral vascularity as well as the prognosis of individuals affected by NSCLC [117]. It has been postulated that TNC is overexpressed at the mRNA and protein levels in tumor tissue from patients with NSCLC [88] and that its degradation may also have biological significance. Specifically, it has been hypothesized that increased degradation of TNC—possibly mediated by elevated MMP-2 activity—may serve as an indicator of recurrence potential and poor prognosis in patients with LC, suggesting a complex role for TNC turnover in tumor progression [118,119].

TNC has been confirmed as a factor that stimulates various cellular processes linked to LC, including the migration and invasion of malignant cells. In addition, its involvement in angiogenesis, a crucial process for the establishment of new blood vessels, a fundamental aspect of neoplastic expansion and dissemination, has been postulated [16,46,47]. For example, the interaction between TNC and α9β1 integrin induces the disorganization of actin stress fibers, leading to the concomitant inhibition of YAP, a regulator of cell motility. This phenomenon favors the development of metastatic processes in the lung [47]. Recent research has revealed that octamer-binding transcription factor 4 (Oct4) exerts inhibitory control over phosphatase and tensin homolog (PTEN) and activates TNC expression through a specific interaction with the transcription factor Sp1. Oct4 represses PTEN in an Sp1-dependent manner by recruiting histone deacetylase 1 and 2 (HDAC1/2) and activating the AKT signaling pathway, which confers drug resistance. In contrast, Oct4 transactivates TNC in an Sp1-independent manner, thereby promoting cancer metastasis. Furthermore, the findings suggest, from a clinical perspective, that in LC patients elevated Oct4 expression, low PTEN levels, and increased TNC expression are correlated with disease progression and poor prognosis [120]. Recent research has suggested that TNC markedly inhibits proliferation triggered by anti-CD3 antibodies and mitogens of human peripheral blood lymphocytes, as well as IFN-γ production by tumor-infiltrating lymphocytes (TILs) isolated from LC specimens. These results indicate that TNC significantly controls the proliferative response and IFN-γ production in LC-associated lymphocytes. In terms of antitumor immunity, the observed inhibition suggests a possible influence of TNC in modulating the immune response against LC, which could imply a negative regulation of the antitumor activity of lymphocytes [88].

Another study analyzed the TME in patients with NSCLC. The results indicated that low levels of CD8+ T cells, low stromal levels of caveolin-1, or high levels of TNC were significant prognostic markers of decreased overall survival. The combination of these factors dramatically increased the risk of disease progression, and the presence of EMT correlated with a reduced immune infiltrate, suggesting possible clinical implications for the management of patients with NSCLC [121]. On the other hand, the available data indicate a positive association between TNC expression and the density of CD31+ and CD34+ blood vessels in NSCLC. These findings suggest the possible involvement of TNC in the process of angiogenesis in the context of NSCLC [16].

This evidence supports the hypothesis that TNC plays a multifaceted role in LC progression, participating in processes such as cell migration and invasion, metastasis, angiogenesis, EMT, immune evasion, drug resistance, as well as tumor progression and poor prognosis. All of this underscores the need for further research to elucidate the molecular and cellular mechanisms underlying LC pathogenesis precisely. Continued research on TNC in this context has the potential to provide valuable information for the diagnosis, prognosis, and treatment of LC. A detailed understanding of its role could lead to the development of more accurate biomarkers for early diagnosis, as well as more targeted therapeutic strategies, thus contributing to a more effective clinical management of this disease.

### 6.3. TNC in Pulmonary Hypertension

PH is a complex and progressive vascular disorder characterized by a sustained increase in pulmonary artery pressure that ultimately leads to right ventricular dysfunction and, in advanced stages, heart failure [122,123]. The classification of pulmonary hypertension encompasses several categories, distinguishing between pulmonary arterial hypertension (PAH), PH associated with left heart disease, PH associated with lung diseases and/or hypoxia, PH associated with chronic pulmonary artery obstruction, and PH with unclear and/or multifactorial mechanisms. The diversity of these categories reflects the complexity of the underlying pathophysiological mechanisms [124]. With respect to the prevalence of PH, contemporary estimates indicate a prevalence of approximately 1% in the global population, with individuals of both genders equally affected, particularly those over 65 years of age. The diagnosis and treatment of this condition pose substantial challenges in the clinical setting, underscoring the urgent need for a deeper understanding of its pathophysiological mechanisms to inform more effective therapeutic strategies. [6,124].

The pathophysiology of PH involves a multifactorial interplay of genetic, molecular, and environmental factors that disrupt the homeostasis of pulmonary artery endothelial cells (PAECs) and pulmonary artery smooth muscle cells (PASMCs) [125,126,127,128]. Several pathogenic pathways involved in the development of PH have been identified. Among these pathways, the nitric oxide (NO) pathway and soluble guanylate cyclase (sGC) play key roles in the regulation of the pulmonary vascular tone. Decreased availability of endogenous vasodilators, such as NO and prostacyclin (PGI2), together with an increase in endogenous vasoconstrictors, such as endothelin (ET-1) and thromboxane, contributes to vasoconstriction and vascular remodeling [127]. In addition, uncontrolled proliferation of pulmonary smooth muscle cells (PSMCs) and increased resistance in pulmonary vessels result in increased pulmonary vascular resistance, which contributes to increased pulmonary arterial pressure [129]. In parallel, persistent inflammation promotes the release of cytokines and growth factors, such as TGF-β, PDGF, VEGF, TNF-α, IL-1β, and IL-6, which stimulate the proliferation of fibroblasts, smooth muscle cells, and resident progenitor cells, contributing to cell proliferation, fibrosis, and vascular obliteration [77,123,129,130]. EMT, induced primarily by TGF-β, not only contributes to myofibroblast accumulation but also promotes excessive ECM deposition in the vasculature and lung parenchyma. In addition, EndoMT represents another important source of myofibroblasts, given that endothelial cells, under profibrotic stimuli such as TGF-β and inflammatory mediators, acquire mesenchymal phenotypes with a high capacity to synthesize collagen and other ECM proteins. Both cellular transitions promote fibrosing remodeling that alters vascular elasticity, increases arterial wall stiffness, and reduces hemodynamic adaptability, processes that together contribute to the establishment and progression of pulmonary hypertension [77,131]. A detailed understanding of the pathogenesis and pathophysiology of pulmonary hypertension is essential for the development of new therapeutic strategies aimed at reversing or modulating the underlying pathological processes.

In the context of PH, the ECM emerges as a crucial component that plays a determining role in the pathogenesis and pathophysiology of this vascular disease. The ECM not only provides structural support for vascular cells but also regulates cell signaling, adhesion, and migration, thus actively influencing the vascular microenvironment [85,123]. In particular, specific ECM components, such as TNC, play prominent roles in the vascular remodeling associated with PH [89,91,123]. Available evidence indicates that the plasma TNC concentration is markedly greater in individuals diagnosed with PH than in their respective controls [91,132]. In individuals affected by familial forms of pulmonary arterial hypertension (PAHF) associated with loss-of-function mutations in bone morphogenetic protein receptor type II (BMPR2), a major genetic cause of PAHF, TNC overexpression is evident in pulmonary vascular lesions. Similarly, PASMCs derived from these patients exhibit increased levels of TNC compared with those derived from healthy individuals [17]. Similarly, a steady increase in TNC mRNA expression has been observed in the lungs and hypertrophied right ventricles (RVs) of rats subjected to monocrotaline (MCT)-induced PH [133,134]. This increase in TNC mRNA expression correlates with an increase in TNC protein level, which is mainly observed in the surrounding parenchyma of muscularized small pulmonary arteries in MCT-treated rats [134]. Similarly, TNC levels are significantly increased in a hypoxia-induced PH model [135]. An investigation of the changes in pulmonary hemodynamics revealed a significant increase in TNC, MMP, and Egr-1 activity in lung tissue during pulmonary artery remodeling compared with control lung tissue. In cell culture, denatured type I collagen promoted TNC expression and Egr-1 activity, whereas native collagen had the opposite effect. These results highlight the key influences of the TNC and MMP on vascular adaptation to hemodynamic changes [94]. TNC has been shown to play a crucial role in the remodeling of pulmonary and systemic arteries by promoting vascular smooth muscle cell (SMC) proliferation, and cellular morphology and TNC expression in SMCs are intrinsically linked to the F-actin cytoskeletal architecture, which is regulated by RhoA and Rho kinase (ROCK); accordingly, ROCK inhibition has been shown to attenuate SMC spreading, ERK1/2 activity, and TNC expression, thereby delaying disease progression in the pulmonary arteries of MCT-induced PH rats [136]. Moreover, Prx1 expression significantly increases SMC proliferation and adhesion. In this context, Prx1 expression has been shown to significantly increase TNC gene promoter activity [137]. Assays performed in a rat model of MCT-induced PH have shown that TNC is upregulated and intimately associated with proliferating cells and that TNC alone or in combination with growth factors such as basic FGF (bFGF) or EGF promotes SMC proliferation [89]. In the context of PH, the available evidence highlights a marked elevation in TNC concentrations. This overexpression of TNC is closely linked to vascular remodeling and SMC proliferation in pulmonary arteries, indicating a crucial role of TNC in disease progression. These results emphasize the importance of the TNC in vascular adaptation to hemodynamic changes, suggesting possible therapeutic targets to address PH and its complications.

### 6.4. TNC in Chronic Obstructive Pulmonary Disease

COPD is a progressive and potentially disabling disease and one of the leading causes of morbidity and mortality worldwide, with an estimated 23% increase in the global mortality rate between 1990 and 2017 [1,138,139]. COPD is a progressive disorder characterized by a sustained inflammatory process that causes narrowing of the airways, resulting in chronic bronchitis and bronchiolitis, as well as damage and destruction of the alveoli, characteristic of emphysema [139,140,141]. The main risk factor is prolonged exposure to inhaled irritants, especially tobacco smoke, although environmental pollution, organic/inorganic dust, and occupational exposures also contribute [139,142].

Prolonged exposure to irritants such as tobacco smoke directly damages the bronchial epithelium, inducing oxidative stress and the release of DAMPs. These alterations promote the early secretion of proinflammatory cytokines (IL-1β, TNF-α) and chemokines (IL-8, CCL2) by epithelial cells [143,144]. This initial proinflammatory microenvironment favors the recruitment and activation of macrophages, neutrophils, and CD8+ T cells, cells that perpetuate inflammation by releasing cytokines (TNF-α, IL-1β, IL-6, IL-8), proteases such as neutrophil elastase and matrix metalloproteinases (MMP-9, MMP-12), as well as ROS [140,141,143,144]. The interaction of these cells with the epithelium and extracellular matrix amplifies tissue damage, leads to the progressive narrowing of the airways, and promotes the destruction of the lung parenchyma [143,144]. This last process manifests clinically as emphysema, which can take different forms. Centrilobular emphysema, the most common, is associated with chronic smoking and predominantly occurs in the upper lobes, manifesting as airspace enlargement in the central region of the secondary lobes, with relative preservation of the distal alveolar structures. Paraseptal emphysema primarily affects the subpleural areas, particularly the upper lobes, and often coexists with fibrosis. In contrast, panlobular emphysema presents as uniform dilation extending from the respiratory bronchioles to the alveoli within the secondary lobule. It is classically described in patients with α1-antitrypsin deficiency, primarily affecting the lower lobes, and may be linked to risk factors other than smoking [145,146].

At the bronchial level, intense remodeling occurs, characterized by goblet cell hyperplasia, squamous metaplasia, and loss of cilia, leading to mucus hypersecretion and impaired mucociliary clearance [143,144]. Furthermore, collagen deposition, peribronchial fibrosis, and bronchial wall thickening reduce the caliber of small airways, leading to progressive airway narrowing. Growth factors such as vascular endothelial growth factor (VEGF), fibroblast growth factor (FGF), and platelet-derived growth factor (PDGF) participate in fibroblast proliferation and extracellular matrix remodeling. Notably, TGF-β not only induces the differentiation of fibroblasts into myofibroblasts and promotes fibrosis, but also promotes EMT in bronchial epithelial cells, contributing to the loss of epithelial integrity, the generation of cells with a mesenchymal phenotype, and the amplification of extracellular matrix deposition [141,143,144,147,148,149,150]. In summary, COPD is the result of a self-perpetuating cycle of chronic inflammation, soluble mediator activation, uncontrolled proteolysis, and structural remodeling, culminating in fixed airway obstruction and progressive deterioration of lung function.

Recently, increasing attention has been given to ECM proteins, particularly to TNC, in the context of COPD. The ECM plays an essential role in the structural integrity of tissues and in modulating key cellular processes. TNC, an ECM protein, has emerged as a central player in tissue remodeling associated with COPD [93,151,152]. Available data suggest that TNC is upregulated in lung biopsies from smokers and COPD patients compared with their respective controls [93,151]. Additionally, patients with severe COPD with left ventricular diastolic dysfunction (LVDD) exhibit different TNC expression patterns because the elevated TNC levels are higher in patients with type II LVDD than in those with type I LVDD, suggesting that TNC levels could be considered a candidate serological biomarker of LVDD in COPD [18].

Studies in a murine model of COPD induced by exposure to fine particulate matter ≤ 2.5 μm in diameter (PM2.5) revealed that exposure to PM2.5 resulted in the development of emphysema, airway wall thickening, increased smooth muscle layer thickness, and decreased lung function, accompanied by a significant increase in the protein levels of Wnt5a, β-catenin, PDGFRβ, and TNC in mouse lung tissue [153]. Furthermore, research has indicated that TNC is positively expressed in primary human airway smooth muscle cells (ASMCs) obtained from COPD patients who are smokers, in contrast to those from nonsmoking patients with COPD, when subjected to stimulation with TGF-β1 [154]. In summary, current evidence indicates a significant association between TNC and the pathophysiology of COPD; however, its direct causal role in disease development and progression has not yet been fully established. Furthermore, the cellular and molecular mechanisms by which TNC modulates pathogenic processes in COPD remain poorly understood, constituting a promising and necessary area of research for future investigation.

### 6.5. TNC in Asthma

Asthma is a chronic inflammatory disease of the airways characterized by reversible airflow obstruction, bronchial hyperreactivity, and recurrent symptoms such as wheezing, dyspnea, chest tightness, and cough [155]. It affects approximately 300 million individuals worldwide, representing a significant public health challenge [155,156]. This heterogeneous condition includes both allergic and non-allergic phenotypes, with variable clinical manifestations and degrees of severity [155].

The pathophysiological cascade of asthma often begins with exposure to environmental triggers such as allergens, pollutants, or respiratory infections—which initiate an immune response [156,157]. Exacerbations of asthma typically involve two phases. The early phase is mediated by allergen-specific IgE bound to mast cells and basophils. Upon re-exposure to triggers such as allergens or pollutants, mast cell degranulation releases histamine, leukotrienes, and prostaglandins, leading to acute bronchoconstriction, mucus hypersecretion, and airway narrowing [155,157]. The late phase occurs hours later, with the recruitment of eosinophils, basophils, neutrophils, and Th2 lymphocytes that sustain inflammation and contribute to ongoing bronchoconstriction. Th2 cytokines (IL-4, IL-5, IL-13) promote IgE production, eosinophil survival, and mucus hypersecretion, while IL-13 and TGF-β play critical roles in airway remodeling and fibrosis [65,155,157].

Persistent inflammation drives airway remodeling, characterized by goblet cell hyperplasia, thickening of the basement membrane, smooth muscle hypertrophy, angiogenesis, and excessive ECM deposition [65,155]. In this context, chronic exposure to mediators such as TGF-β can induce EMT, whereby epithelial cells lose polarity and acquire mesenchymal features, while fibroblasts differentiate into myofibroblasts. These processes promote subepithelial fibrosis, airway wall thickening, and progressive airflow limitation. Vascular hyperplasia and increased permeability of the lamina propria further amplify acute bronchoconstrictive episodes [65,66,155,158]. Although asthma typically begins as a fully reversible disease, long-standing airway inflammation and remodeling may lead to partially irreversible obstruction, explaining why some patients develop fixed airflow limitation with disease progression.

The ECM, in the context of asthma, acts as a dynamic environment that modulates cellular function and the inflammatory response. TNC has emerged as a key player in these processes [95,159]. Recent research has revealed that the TNC levels are markedly greater in the airways of patients with asthma than in those of control subjects [92]. Furthermore, the serum levels of TNC are increased in asthma patients, and its expression is correlated with disease severity [20,160]. Studies in a murine model of asthma induced by sensitization and challenge with ovalbumin revealed that TNC^−/−^ mice are protected from inflammation, and the number of eosinophils in the lung compared to WT mice, airway hyperreactivity, NF-κB activation, and monocyte chemoattractant protein-1, IL-5, IL-13, MMP-9, and Ig-E concentrations in bronchoalveolar lavage fluid are significantly decreased in TNC^−/−^ mice [95]. Moreover, a study in an ovalbumin-induced murine model of asthma revealed that, compared with WT mice, STAT4^−/−^ mice presented low TNF-α and IFN-γ mRNA expression, resulting in a significant reduction in TNC expression in the airways. These results suggest that TNC expression is regulated by the transcription factor STAT4, TNF-α, and IFN-γ in allergic airway inflammation [161]. TNC has been reported to induce MMP-1 expression in ASMCs through the activation of ERK1/2, JNK, and p38 MAPK. MMP-1 is a collagenase that is upregulated in patients with asthma, and its expression is potentially associated with airway narrowing and asthma symptoms [159]. Available evidence suggests that stimulating BEAS-2B cell line or primary human bronchial epithelial (HBE) cells with TGF-β1 significantly increases TNC mRNA levels in a dose-dependent manner, suggesting that TGF-β1, an important cytokine in the development of asthma, may induce TNC expression to contribute to the development of chronic asthma [162].

Additionally, rhinovirus infection, a common cause of asthma exacerbation, has been suggested to promote TNC expression and release in HBE cells [163]. Similarly, one study showed that primary HBE cells isolated from asthma patients secreted significantly more TNC than did the respective controls. Furthermore, the TNC expression levels increase significantly in HBE cells from asthma patients and healthy patients in response to a mechanical compression stimulus that mimics bronchospasm during asthma exacerbation, and TNC production and release were shown to be dependent on the ERK and TGF-β receptor pathways [15]. Surprisingly, these studies revealed that rhinovirus infection and mechanical compression stimulation promoted an increase in the expression of TNC transported in EVs released by primary HBE cells, suggesting that the essential role of TNC in asthma-related processes and complications may be closely related to its release as part of the EV cargo, a novel and understudied cellular communication mechanism in this disease [15,163]. Therefore, the available data suggest that TNC may play an essential role in the development of asthma, given that it has been linked to lung inflammation and airway remodeling. Thus, it emerges as a promising candidate for future research to more precisely elucidate its role in this disease.

## 7. Conclusions

CRDs, such as asthma, COPD, lung cancer, PH, and IPF, exhibit marked heterogeneity in their epidemiology, pathogenesis, and clinical manifestations. However, they also share fundamental pathophysiological mechanisms, such as chronic inflammation, airway or parenchymal remodeling, ECM dysregulation, and aberrant epithelial–mesenchymal interactions.

In these diseases, the ECM emerges as a dynamic and central player, not only as a structural scaffold but also as a bioactive regulator of cell signaling, tissue remodeling, and inflammatory responses. In this context, TNC, a matricellular glycoprotein overexpressed in tissue injury and remodeling, emerges as a potentially relevant molecule. Available evidence indicates that TNC is involved in processes such as EMT induction, fibroblast activation, immune response modulation, and promotion of an invasive phenotype in cancer. While the level of evidence supporting the role of TNC varies across these diseases, being more established in cancer and fibrotic diseases and more limited in others, such as PH, the recurrent involvement of ECM dysregulation suggests that TNC could act as a convergent element in their progression.

Considering these observations, TNC could represent both a common mediator and a contextual modulator of the pathogenesis of CRDs. In asthma and COPD, TNC could contribute to airway remodeling through ECM–cell interactions and proinflammatory signaling. In LC, TNC may facilitate tumor progression within a remodeled ECM. In IPF and PH, TNC may amplify fibrotic or vascular remodeling cascades through sustained mesenchymal activation. These hypotheses warrant further investigation, as a deeper understanding of the roles of TNC could provide new insights into shared and disease-specific therapeutic targets in CRDs.

## Data Availability

Not applicable.
